# From Lab to Field: The Influence of Urban Landscapes on the Invasive Potential of *Wolbachia* in Brazilian *Aedes aegypti* Mosquitoes

**DOI:** 10.1371/journal.pntd.0003689

**Published:** 2015-04-23

**Authors:** Heverton Leandro Carneiro Dutra, Lilha Maria Barbosa dos Santos, Eric Pearce Caragata, Jéssica Barreto Lopes Silva, Daniel Antunes Maciel Villela, Rafael Maciel-de-Freitas, Luciano Andrade Moreira

**Affiliations:** 1 Mosquitos Vetores: Endossimbiontes e Interação Patógeno Vetor, Centro de Pesquisas René Rachou—Fiocruz, Belo Horizonte, Minas Gerais, Brazil; 2 Laboratório de Transmissores de Hematozoários, Instituto Oswaldo Cruz, Fiocruz, Rio de Janeiro, Rio de Janeiro, Brazil; 3 Programa de Computação Científica, Instituto Oswaldo Cruz, Fiocruz, Rio de Janeiro, Rio de Janeiro, Brazil; University of Perugia, ITALY

## Abstract

**Background:**

The symbiotic bacterium *Wolbachia* is currently being trialled as a biocontrol agent in several countries to reduce dengue transmission. *Wolbachia* can invade and spread to infect all individuals within wild mosquito populations, but requires a high rate of maternal transmission, strong cytoplasmic incompatibility and low fitness costs in the host in order to do so. Additionally, extensive differences in climate, field-release protocols, urbanization level and human density amongst the sites where this bacterium has been deployed have limited comparison and analysis of *Wolbachia*’s invasive potential.

**Methodology/Principal Findings:**

We examined key phenotypic effects of the *wMel Wolbachia* strain in laboratory *Aedes aegypti* mosquitoes with a Brazilian genetic background to characterize its invasive potential. We show that the *wMel* strain causes strong cytoplasmic incompatibility, a high rate of maternal transmission and has no evident detrimental effect on host fecundity or fertility. Next, to understand the effects of different urban landscapes on the likelihood of mosquito survival, we performed mark-release-recapture experiments using *Wolbachia*-uninfected Brazilian mosquitoes in two areas of Rio de Janeiro where *Wolbachia* will be deployed in the future. We characterized the mosquito populations in relation to the socio-demographic conditions at these sites, and at three other future release areas. We then constructed mathematical models using both the laboratory and field data, and used these to describe the influence of urban environmental conditions on the likelihood that the *Wolbachia* infection frequency could reach 100% following mosquito release. We predict successful invasion at all five field sites, however the conditions by which this occurs vary greatly between sites, and are strongly influenced by the size of the local mosquito population.

**Conclusions/Significance:**

Through analysis of laboratory, field and mathematical data, we show that the *wMel* strain of *Wolbachia* possesses the characteristics required to spread effectively in different urban socio-demographic environments in Rio de Janeiro, including those where mosquito releases from the Eliminate Dengue Program will take place.

## Author Summary

*Wolbachia* are maternally inherited bacterial endosymbionts that induce cytoplasmic incompatibility in mosquitoes, and use this pattern of sterility to facilitate their spread into naïve, uninfected host populations. These bacteria were recently introduced into *Aedes aegypti*, a species naturally free of *Wolbachia*, and the primary vector of dengue virus. *Wolbachia* are currently being used as a biological tool to control dengue transmission in many countries, including Brazil. We performed a series of laboratory and field assays and then created mathematical models in order to understand the invasive ability of the *w*Mel *Wolbachia* strain in Brazilian *Ae*. *aegypti* mosquito populations. We show that *w*Mel infection had no detrimental fitness effects on its new Brazilian host, and would theoretically be able to successfully invade the mosquito populations in the five distinct urban landscapes that we studied. We found that *Wolbachia* spread more easily where the local mosquito population was smaller. The size of the local human population did not overtly influence the likelihood of spread. These findings provide insight into how the nature of different urban landscapes will affect the likelihood of *Wolbachia* spreading effectively, which will greatly assist future releases of *Wolbachia*-infected mosquitoes in Brazil as part of the Eliminate Dengue Program.

## Introduction

Dengue is widely considered the most important vector-borne virus, with an estimated 2.5 billion people living at risk of infection in more than 100 countries [1]. The virus is transmitted by mosquitoes from the genus *Aedes*, with *Aedes aegypti* being the primary vector. There are an estimated 100 million new cases of the disease each year, and this imposes a heavy burden on global health and economics, with the average annual cost of the disease estimated to be US$2.1 billion for the Americas alone [2]. Control efforts are complicated by the current lack of an effective dengue vaccine, the emergence of insecticide resistance in natural vector populations, and the difficulties and costliness associated with maintaining effective suppression of mosquito breeding sites. Consequently, alternative approaches are required in order to restrict dengue transmission [3].

A novel and promising form of biocontrol designed to reduce dengue transmission utilizes the endosymbiotic bacterium *Wolbachia pipientis*, which is naturally present in up to 40% of all insect species [4]. Different strains of this bacterium can cause a variety of phenotypic effects. Of these, the phenotypes of cytoplasmic incompatibility (CI), high bacterial density, and as a result, a high maternal transmission rate are the two main determinants of *Wolbachia*’s “drive” mechanism, which facilitates the spread of the bacterium into field mosquito populations [5]. CI is a reproductive incompatibility that prevents females without *Wolbachia* from producing viable offspring after copulation with *Wolbachia*-infected males. In contrast, *Wolbachia*-infected females can successfully reproduce after mating with any male. This increases the frequency of *Wolbachia* infection in a given population with each subsequent generation. *Ae*. *aegypti* is not naturally infected with *Wolbachia*, but has been transinfected with several different *Wolbachia* strains that cause strong CI [6–8]. A further, significant discovery was the fact that some *Wolbachia* strains can prevent or severely restrict infection by key pathogens, including dengue virus, in the host mosquito [7,9]. Together these phenotypes would allow the replacement of wild, dengue-susceptible mosquitoes with dengue-resistant, *Wolbachia*-infected mosquitoes potentially ameliorating the great burden of dengue [10].

The use of *Wolbachia* as a biocontrol tool to reduce dengue transmission is currently being tested in several countries (www.eliminatedengue.org). Here *Wolbachia*-infected mosquitoes are released in large numbers over a period of weeks, and eventually replace the wild *Ae*. *aegypti* population. So far, field trials have already been conducted in four sites in Australia, one in Vietnam, and two in Indonesia, while trials in Brazil have been underway since September 2014 using *Ae*. *aegypti* infected with the *w*Mel *Wolbachia* strain. The Brazilian trials are set to take place at four different field sites over the next two years. Critically, there is a great deal of variability in the physical, environmental and socioeconomic conditions between these four sites, and between those from the other countries involved in the project. These differences include factors such as climate, housing and population density, sanitation conditions and the use of piped or stored drinking water. All of these factors can affect the size and characteristics of the already-established wild mosquito populations [11], which in turn determines the number of *Wolbachia*-infected mosquitoes that must be released in order for the bacterium to spread to high frequencies in a given area [12]. As such, it is important to identify these factors and determine if any could potentially limit the invasive potential of *Wolbachia*, as this could necessitate that mosquito release characteristics, including release duration and mosquito numbers, be tailored for individual release sites within a city.

Therefore, the objective of this work was to determine whether the likelihood of a successful *Wolbachia* invasion could be influenced by differences between release sites, in preparation for the release of *Wolbachia*-infected *Ae*. *aegypti* in Brazil. To address this objective, we first examined whether the key invasion phenotypes of CI and maternal transmission were present in *Ae*. *aegypti* with a Brazilian genetic background, infected with the *w*Mel *Wolbachia* strain, as these phenotypes had only previously been characterised in mosquitoes from other genetic backgrounds. We then investigated the characteristics of *Ae*. *aegypti* populations in five Brazilian suburbs in Rio de Janeiro with different socio-demographic characteristics. Of special interest were those factors that could directly influence or restrict the spread of *Wolbachia* into natural mosquito populations, including the *Ae*. *aegypti* population size, and mosquito daily survival rate. Finally, by amalgamating these data we were able to simulate the likelihood that *Wolbachia* would disseminate and spread so that all mosquitoes at the five sites became infected. We observed that successful invasion would likely occur for all sites; however the means by which is did so varied between the sites, and invasion occurred more slowly at sites where local mosquitoes were more abundant. Through the results of these models we were then able to develop optimized release strategies for each field site, which will greatly benefit the upcoming biocontrol trials, and likely prove applicable to field sites in other cities and countries that share similar characteristics.

## Methods

### Ethics statement

MRR experimental protocols were submitted to and approved by FIOCRUZ Ethical Committee (CEP/FIOCRUZ protocol no. 22286313.7.0000.5248). Permission was obtained from the Rio de Janeiro Department of Health before releases commenced. Local residents received a full explanation of the project by at least one of the co-authors, and by the same health agent who visited their home 4–6 times per year during routine mosquito surveillance. This explanation highlighted the relevance and objectives of the MRR experiment.

Households were only entered after receiving formal written consent from the householders.

### Laboratory assays

#### Mosquito rearing

All mosquitoes used in these experiments were maintained in a climate controlled insectary, at 27 ± 1°C and 70 ± 10% relative humidity, with a 12:12 hour light:dark cycle. Larvae were reared in filtered and dechlorinated water, and fed with one Tetramin Tropical Fish Food tablet every two days. Adult mosquitoes received 10% sucrose solution ad libitum, and females were blood-fed weekly with human blood using a Hemotek membrane feeder (Hemotek Ltd) when eggs were required for experimental or colony rearing purposes. Prior to feeding, the blood was tested for the presence of dengue virus using the Dengue NS1 Ag Strip test (BioRad Laboratories).

#### Backcrossing of *w*Mel-infected *Ae*. *aegypti*

*w*Mel-infected *Ae*. *aegypti* were imported to Brazil from Australia (IBAMA license 11BR005873/DF). Prior to field releases in Brazil it was necessary to create a *Wolbachia*-infected line with a Brazilian genetic background. To that end, the Australian *w*Mel-infected mosquitoes were backcrossed with wildtype Brazilian mosquitoes collected from four different districts in Rio de Janeiro (Paquetá, Jurujuba, Belford Roxo and Vila Valqueire) following a previously described protocol [13]. The parental generation in the backcross consisted of 250 virgin, *Wolbachia*-infected, Australian females and 200 uninfected, Brazilian males. This process was repeated for nine generations in order to generate the backcrossed *Wolbachia*-infected line named *w*Mel_Br.

#### Generation of *Ae*. *aegypti* line cured of *w*Mel infection

A group of *w*Mel_Br mosquitoes were cured of their *Wolbachia* infection through treatment with antibiotics over three successive generations. Adult mosquitoes were provided with 10% sucrose solution containing 0.1mg/mL of tetracycline hydrochloride for a period of 10 to 14 days, which encompassed the first gonotrophic cycle [14]. Approximately 1000 adult mosquitoes were treated every generation, in order to limit detrimental genetic effects that might occur as a consequence of low population size. In each generation, twenty-four females and twelve males were randomly screened using qPCR (as described below) to confirm the loss of *Wolbachia*. Antibiotic treatment was discontinued when 100% of samples were negative for *Wolbachia*. This process took three generations to complete. The resulting line, named *w*Mel_BrTET, was allowed two generations for microbiota reacquisition prior to the start of experiments. During this period, *w*Mel_BrTET larvae were reared in trays dosed with the larval water from wildtype mosquitoes that were not treated with antibiotics.

#### Monitoring *Wolbachia* infection status

Conventional PCR for the *Wolbachia surface protein* (*wsp*) gene was used to examine the *Ae*. *aegypti* colonies for the presence of *Wolbachia* [15]. DNA was extracted from individual mosquitoes by homogenization in 80μL of Squash buffer using a Mini-Beadbeater-16 (BioSpec) [16]. PCR reactions were performed using a Veriti Thermal cycler using previously published primers for *wsp* and the *Ae*. *aegypti* Ribosomal S17 (*rps17*) gene [9], following previously described reaction conditions [14]. In cases where the PCR result was unclear, samples were rerun using real-time PCR on a 7500 Real-Time PCR System (Life Technologies). For real-time PCR, crude DNA was extracted from individual mosquitoes according to existing protocols [16], and then quantified in duplicate for both *wsp* and *rps17*, using a two-step reaction (Vol. = 10μL) using 2X SYBR Green PCR Master Mix (Applied Biosystems) [14].

#### Cytoplasmic Incompatibility and maternal transmission

**Fig 1:**
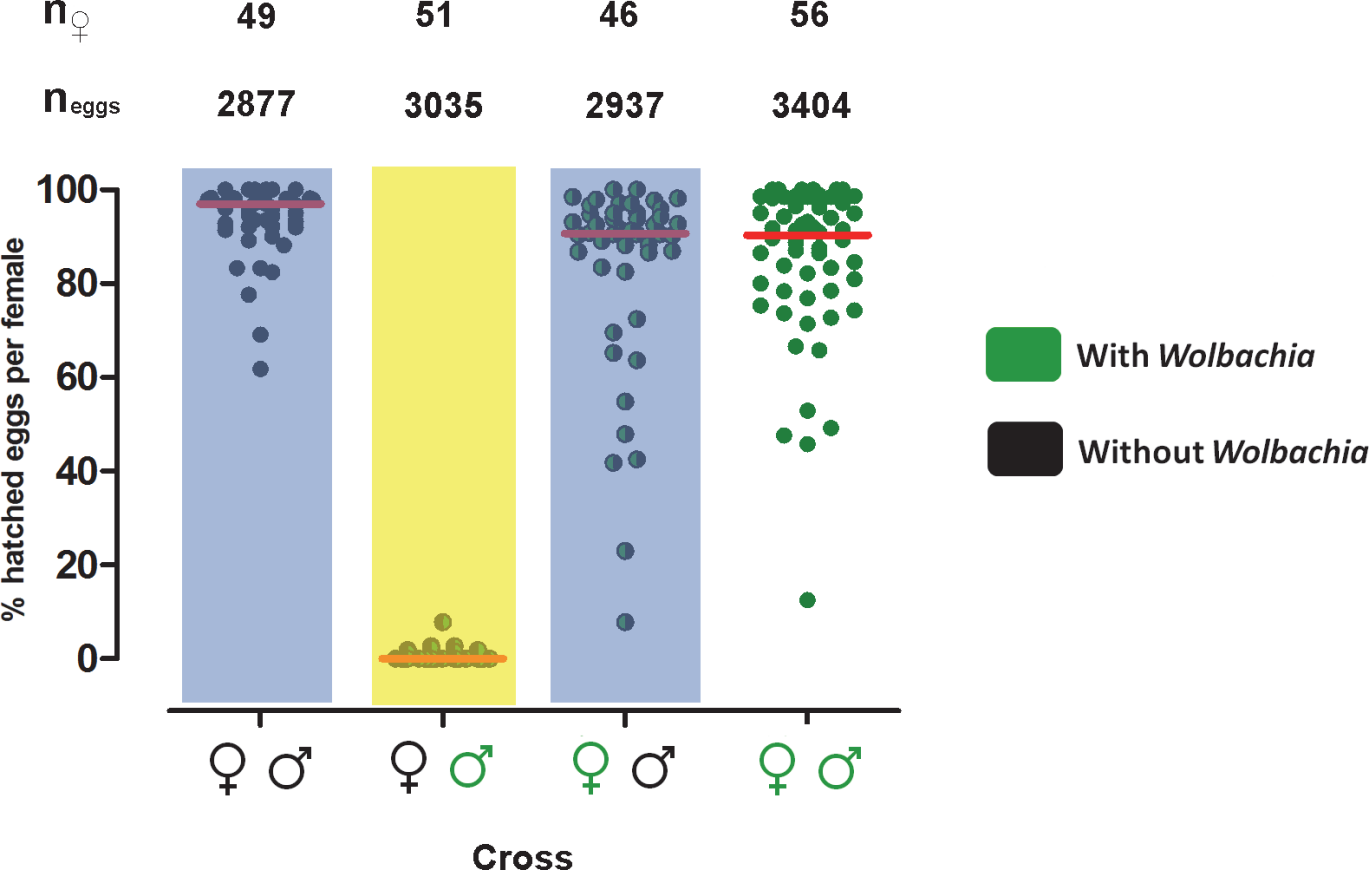
*w*Mel causes strong cytoplasmic incompatibility in Brazilian *Ae*. *aegypti* mosquitoes. Percentage of hatched eggs per female for all potential crosses between *w*Mel-infected Brazilian *Ae*. *aegypti* (green) and *Wolbachia*-uninfected Brazilian *Ae*. *aegypti* (black). Each circle represents a single adult female, while the red horizontal lines indicate the median number of hatched eggs for each cross. The cross highlighted in yellow is the incompatible cross and was statistically different from the other three groups, while the two crosses highlighted in blue had significantly different hatch rates according to Dunn’s test. These data were pooled from two independent biological replicates. The total number of females (n_♀_) and the total number of eggs (n_eggs_) are indicated above each cross.

To determine if *w*Mel caused CI in the Brazilian genetic background, reciprocal crosses were performed between *w*Mel_Br and *w*Mel_BrTET lines. In two replicate experiments, 50 male and female pupae from each line were sexed by visual analysis of their terminalia using a stereomicroscope, and then placed in a single cage (each cage representing one of the reciprocal crosses depicted in Fig 1) where they were allowed to mate for five days. On the fifth day, females from all groups were blood-fed for one hour using a Hemotek feeder. At 72 hours post-feeding, thirty females from each group were randomly selected and placed individually in inverted petri dishes containing moist filter paper for oviposition [17]. The total number of eggs laid by each female (fecundity) was counted using EggCounter V 1.0 [18]. The number of hatched eggs from each female (fertility) was counted with the aid of a stereomicroscope. Conventional PCR for the *wsp* gene (as described above) was used to check the infection status of males and females in all reciprocal crosses. A TaqMan-based assay was used for verification when conventional and SYBR Green-based real-time PCR results were unclear. In order to evaluate the rate of *Wolbachia* maternal transmission, females originally used in the cytoplasmic incompatibility experiment described above, had their eggs hatched and their offspring reared and tested for the presence of *Wolbachia*. Total DNA from larvae, pupae and adults was extracted and then used in the conventional PCR assay for *wsp* (as described above).

### Field assays

#### Study areas

For our field release assays, we selected five distinct field sites in the vicinity of Rio de Janeiro, Brazil. Collectively, these sites are representative of the broad range of socio-demographic and economic conditions found across the city, with each providing a distinct environment for mosquito release (Table 1). Demographic data from all five of the sites were collected from the 2010 Census (http://censo2010.ibge.gov.br/). The remaining descriptive data were collected via observation during the field site characterization, and during previous experiments [19,20].

**Table 1:** Field site characteristics.

	Field Site				
Characteristic	Amorim	Jurujuba	Tubiacanga	Vila Valqueire	Urca
Co-ordinates	22°52'29"S; 43°14'54"W	22°55'37"S; 43°07'11"W	22°47'06"S; 43°13'32"W	22°53’17” S; 43°22’20” W	22°56’43” S; 43°09’42” W
Surrounding environment	Highways & vegetation	Guanabara Bay	Guanabara Bay	Secondary forest	Sugar Loaf Mountain
Socio-demographic description	Favela (slum)	Working class	Lower middle class	Middle class	Upper middle class
Paved Streets	No	Partially	Partially	Yes	Yes
Piped water	No	No	Some	Yes	Yes
Garbage collection	No	No	Yes	Yes	Yes
External water tanks	Yes	Yes	Yes	Yes	No
Common mosquito habitats	Water tanks and metal drums	Water tanks and metal drums	Water tanks and metal drums	Water tanks and buckets	Domestic drains and abandoned plastic pots
Number of houses	897	399	753	410	1284
Housing type	Very small	Small	Standard	Large (3–4 bedrooms)	Large duplexes (at least 3 bedrooms)
Inhabitants	2992	1196	2425	1423	3212
Human density (inhabitants/ha)	1086.03	340.2	165.98	74.17	234.54
Site size (square blocks)	10	6	14	15	13
Area (ha)	2.755	3.515	1.461	19.185	13.695

#### Characteristics of mosquito populations at field sites

Approximately 30 BG-Sentinel traps (Biogents) were deployed at each of the five field sites in order to collect *Ae*. *aegypti* females, as a means to estimate the mosquito population size, as well as the species composition at each field site. The traps were checked weekly between October 2012 and October 2014, for a total of 104 weeks of mosquito collection. Each trap was analysed individually. All captured mosquitoes were transported to the laboratory, mosquito numbers were counted and individual mosquitoes were identified down to the species level using taxonomic keys [21].

#### Probability of daily survival

In order to estimate mosquito survival we conducted mark-release-recapture experiments in Jurujuba, a working class area, and Vila Valqueire, a middle class area. Information regarding the probability of daily survival (PDS) from the other three field release was obtained during previous MRR experiments [19,22]. The mosquitoes used in these experiments were the F1 and F2 generations of eggs collected from 40 ovitraps that were deployed at the field sites. These mosquitoes were reared as described above, unless specified below. Wildtype mosquitoes were used in these assays in order to obtain an accurate picture of the fitness of mosquitoes already present at the sites where *Wolbachia*-infected mosquitoes will be released.

#### Mark and release

Before each experiment, eggs were divided into two groups, which were hatched 3 days apart to produce two independent cohorts of adult females. Females from one cohort were blood-fed approximately 18h before release (Cohort A), whereas females from the second cohort were released while unfed (Cohort B). The reason for this disparity was to observe potential effects on the PDS due to blood feeding. A greater PDS amongst Cohort A mosquitoes could promote more effective invasion by *Wolbachia* as the mosquitoes would not need to spend time searching for a host. We sought to assess whether there were any benefits to releasing using this technique. Each cohort was marked with a different colour fluorescent powder (Day-Glo Color Corp.) in small cylindrical cups (12 x 10cm). Females from both cohorts were released into the test sites on the morning of their fourth day post-eclosion (between 8:00 AM and 9:00 AM), 1h after powder application. Mosquitoes were released outside of the houses at the field sites, with an average of 4.2 mosquitoes/house released in Vila Valqueire and 7.5 in Jurujuba.

#### Recapture

Marked females were captured with CDC backpack aspirators (John W. Hock), and sticky traps [20]. Captures started one day after mosquito release. Fifteen houses were randomly selected per day for backpack aspiration. Collections were completed within 15–20 mins for each house. The entire property, inside and outside, was aspirated. If the house in question had a backyard or garden, mosquitoes in that area were also collected, but only within the limits of the property. Sticky traps were installed in 15 randomly selected houses. These were monitored daily for the presence of dust-marked females, throughout the course of the assay. Occasionally, aspiration was conducted in the same house where a sticky trap was installed. Daily captures were conducted by nine consecutive days. Captured mosquitoes were examined and sorted by cohort/powder type under UV light (S1 Table).

#### Climatic conditions during MRR assays

The Climate in Rio de Janeiro is characterized by a dry winter (May–August) and a wet summer season (November–March). MRR experiments were performed during September (Vila Valqueire) and November (Jurujuba). During the thirteen days of fieldwork (one day to release and twelve of capturing marked mosquitoes), we registered a mean temperature of 25.7 ± 4.8°C, 6.8 mm rainfall, mean relative humidity of 64.54% and an average wind speed of 1.67m/s in Vila Valqueire. While in Jurujuba there was a mean temperature of 24.7 ± 2.6°C, 55.8mm total rainfall, mean relative humidity of 81.88%, and an average wind speed of 2.31m/s. Air temperature and precipitation data for these periods were recorded every hour and were obtained from meteorological stations located less than 10km away from the two study areas.

#### Survival rate analysis

PDS for each of the two cohorts was estimated by fitting two models, the first being exponential [23], and the second, nonlinear [24]. From these models we derived two values: the average life expectancy (ALE), defined as 1/**–**log_e_ PDS, and longevity, defined as PDS^10^ (where 10 is the estimate of the duration of the extrinsic incubation period for dengue in days given the temperature of the field sites) [25], which gave us the expected proportion of mosquitoes surviving long enough to transmit dengue virus. We fit both models to our data, using linear and nonlinear least squares standard procedures using R version 3.0.1 [26].

### Modelling the invasive potential of *w*Mel in the five field sites

#### Abundance estimation

We used a logistic regression model based on a Lincoln type index to estimate the size of the *Ae*. *aegypti* population at each of the five field sites. This model incorporated the PDS estimations calculated from the MRR experiments and also demographic data from each field site (using two parameters; (1) the number of premises, and (2) number of inhabitants). The number of captures of marked mosquitoes (observations) was modeled as a binomial variable “*y*_*i*_ ~ Bin(*N*_*i*_, *p*_*d*_)”, where “*p*_*d*_” is the probability of capture, and “*N*_*i*_” is the number of mosquitoes released at each site. We assumed that the probability of capture in the MRR experiments varied depending on each site's conditions, namely the number of people living in each area (*hpopsize*) and the number of premises in the area (*premises*), as represented by a logistic model:

$$\text{logit}\left(p_{d}\right)\sim \;k_{0}+k_{1}\;\times \;premises+k_{2}\;\times \;hpopsize$$

where “logit” represents a logistic function. The coefficient *k*_*0*_ describes the proportion of the mosquito capture rate that is associated with intrinsic characteristics of the type of trap used. The coefficients *k*_*1*_, and *k*_*2*_ indicate the weighting of each of the two variables (number of premises and human population size) indicating the extent to which each variable influenced the probability of mosquito capture. These coefficients were estimated using the GLM framework in the R statistical platform. These two variables were included in the model because they both had a statistically significant influence on the likelihood of mosquito capture (at the 95% significance level), and because a two-variable model was a better fit for our MRR data, than compared to models that included additional variables (geographical area, human density). These other variables did not have a significant effect on mosquito capture. This type of modeling approach differs to the more traditional use of a Lincoln Peterson estimation, which considers all variables for each site as a single coefficient. Although there were clear landscape heterogeneities between the sites, the recapture protocol we used was identical, which meant that the significance of these parameters could be estimated using logistic regression [27]. The estimate of mosquito abundance at each field site was described by the relationship between the average number of mosquitoes collected each week and their recapture probability. These values were derived from the model and adjusted for weekly differences, and then used to represent the carrying capacity of each site in the mathematical models used to examine *w*Mel invasion (see below).

#### Invasion analysis

In order to study the spread of *Wolbachia* into the five field sites, we used models based on ordinary differential equations (ODE) [28] to mathematically describe the dynamics of the invasive potential of the *w*Mel strain. The models simulated 12 weekly releases of a group of *w*Mel-infected mosquitoes into each of the field sites (the size of this group varied in order to determine the number of mosquitoes required for successful spread), and then described changes to the proportion of *Wolbachia*-infected individuals in the total mosquito population over time. We assumed that the female-to-male ratio was equal for both populations [28]. These models incorporated parameters derived from our phenotypic characterization of *w*Mel-infected Brazilian mosquitoes, and characteristics of the local mosquito population at each field site (Table 2). In this model *Wolbachia* infection imposed a fitness cost, increasing the mortality of infected individuals [11].

**Table 2:** Field site mosquito population characteristics & model parameters.

**Field Site Characteristics derived from MRR experiments**					
	**Vila Valqueire**	**Tubiacanga**	**Jurujuba**	**Urca**	**Amorim**
Number of mosquitoes released	1730	4572	3000	1750	4220
Number of recaptured mosquitoes	98	478	93	171	512
Weekly Probability of capture	0.0264	0.0661	0.0214	0.0572	0.0696
Carrying capacity (K)	9705	2993	8499	2518	1084
Probability of daily survival (PDS)	0.73	0.73	0.81	0.65	0.93
**ODE Model parameters used in *w*Mel invasion scenarios**					
**Parameter**		**Symbol**		**Value**	
Probability of cytoplasmic incompatibility		*c*		0.9951	
Probability of eggs developing to adulthood		*p*		0.02	
Egg output rate		*α* (day^-1^)		60	
Probability of vertical transmission of *w*Mel		*v*		0.96, 0.99	
Life shortening rate of *w*Mel		*s*		0.10, 0.33	

The number of uninfected mosquitoes was estimated from data from field experiments. The probability of eggs developing to adulthood was derived from the field data, including PDS estimates. All other data were derived from laboratory experiments

The two ODE were as follows:

$$\frac{dI}{dt}=v.b.I-\;\frac{b\;\left(1+s\right)I\;\left(I+U\right)}{K}$$ 1

$$\frac{dU}{dt}=b.f.\;\left(U+(1-v\right)I)-\frac{b\;\left(I+U\right)U}{K}$$ 2

Where Eq 1 describes the *w*Mel-infected population, and Eq 2 the-uninfected, wildtype population. The variables *I* and *U* respectively describe the number of infected and uninfected individuals. The equations contained several parameters describing the release and spread of *Wolbachia*. *v*—the vertical transmission rate of *w*Mel. *b*—the recruitment rate (*b*=log(1+*α*.*p*)), where *α* is the mean number of eggs laid, and *p* is the probability that any given egg would result in a live adult mosquito. The value for *p* was derived from the PDS data from each field site. *s*—the life shortening effect associated with *w*Mel infection. *K*—the carrying capacity of the mosquito population at each field site, as calculated using the logistic regression models. *f*—the cost of CI in the uninfected population, $f=1-\frac{c.I}{\left(I+U\right)}$, where *c* is the rate of CI.

For each site, we considered scenarios involving changes to the maternal transmission rate and decreased longevity resulting from *w*Mel infection in order to determine their effect on the likelihood of *Wolbachia* spreading to infect all mosquitoes in the area. We then considered changes to the number of *Wolbachia*-infected mosquitoes released each week, in order to determine the optimal release strategy at each site.

### Data analysis

The CI data were analysed using Kruskal-Wallis one-way analysis of variance, followed by pairwise comparison using Dunn’s tests, and fecundity data were compared using Mann-Whitney U-tests. Differences in survival rates between the field sites were evaluated using exponential and non-linear models. Exponential models have traditionally been used to estimate *Ae*. *aegypti* survival but these possesses two fundamental drawbacks; they assume a priori that mosquito mortality does not vary with increasing age, and do not account for the removal of mosquitoes from the population during the recapture process. Consequently, we utilised a non-linear survival model, which incorporated a correction for the removal of individuals [24]. All results were analysed through GraphPad Prism version 5.03 for Windows (GraphPad Software, CA, (www.graphpad.com), or using R 3.0.1 software [26].

## Results

### CI and fecundity

**Fig 2:**
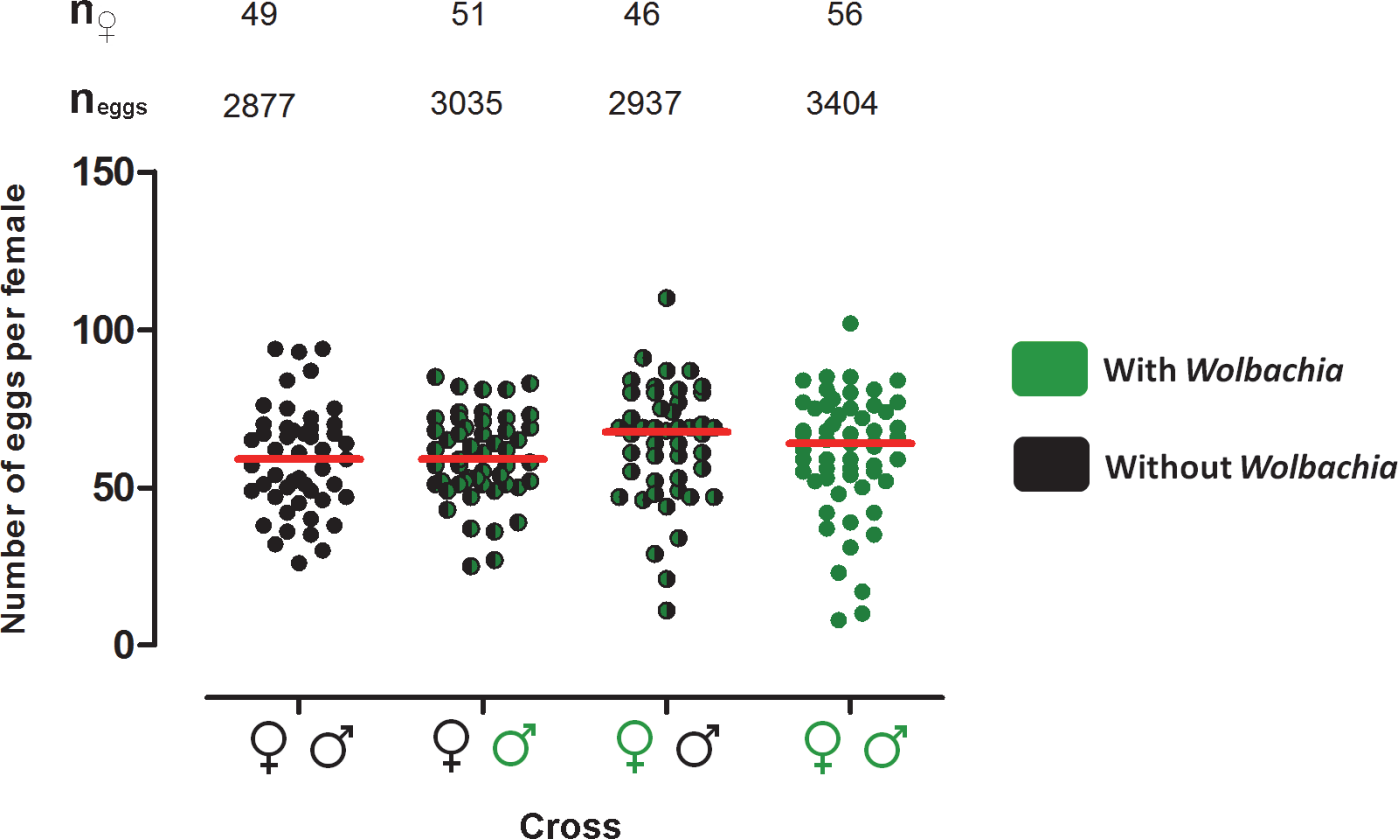
*w*Mel does not affect fecundity in Brazilian *Ae*. *aegypti*. Number of eggs laid by females from the reciprocal crosses between *w*Mel-infected (green) and antibiotic-treated (black) Brazilian *Ae*. *aegypti* mosquitoes. Each circle represents a single adult female, while the horizontal red lines indicate the median number of eggs laid in each cross. The presented data are pooled from two independent biological replicates. No significant differences were detected between the four crosses. The total number of females (n_♀_) and the total number of eggs (n_eggs_) are indicated above each cross.

Reciprocal crosses between the *w*Mel_Br and *w*Mel_BrTET mosquito lines revealed strong cytoplasmic incompatibility due to the presence of *w*Mel in *Ae*. *aegypti* mosquitoes with a Brazilian genetic background (Fig 1). A CI rate of 99.51% was observed when uninfected females were crossed with infected males (the “incompatible” cross), where only 15 of 3035 eggs hatched, a significantly lower proportion than what was observed in the other crosses (Kruskal-Wallis, *H* = 124.1, *df* = 3, *P <* 0.0001). Conversely, the median percentage egg hatch per female in the other three “compatible” crosses ranged between 90.3 and 96.9%. Additionally, the cross between infected females and uninfected males resulted in a higher number of hatched eggs compared to the cross between uninfected females and uninfected males (Mann-Whitney U test, *U* = 603.5, *df* = 1, *P <* 0.0001). All females involved in the three compatible crosses laid at least one viable egg, indicating that they all had mated successfully. Although there was slightly higher fecundity in crosses involving *w*Mel*-*infected females, there were no significant statistical differences between the four experimental crosses (Fig 2; Kruskal-Wallis, *H* = 4.141, *df* = 3, *P* = 0.2466).

### Maternal transmission rate

All adult *w*Mel_Br females that were involved in the first replicate of the CI experiments were screened for *Wolbachia* infection using qPCR, with a 100% infection rate observed. The resulting progeny from these mosquitoes were also screened for *Wolbachia* to provide an estimate of the maternal transmission rate in the Brazilian genetic background. Overall, 757 larvae, pupae and adult mosquitoes were screened. Of these, 725 were infected and 32 were uninfected, which implied a 96% maternal transmission rate of *w*Mel, in Brazilian *Ae*. *aegypti* mosquitoes. This experiment was then repeated using the progeny from the second CI experiment. This time only adult mosquitoes were screened for *Wolbachia*, and 239 of 239 samples were identified as positive, indicating a 100% maternal transmission rate.

### Mosquito population characteristics at field sites

Over the course of the two-year collection period we collected a total of 181,937 wild mosquitoes across the five field sites; 82,316 of these were *Ae*. *aegypti*, 2,867 were *Aedes albopictus* and 96,754 were *Culex quinquefasciatus*. We observed differences in the mean weekly number of mosquitoes captured between the sites (Table 3). The highest numbers of collected mosquitoes were obtained from Vila Valqueire, the middle class area, where 6043 more mosquitoes were collected than in Tubiacanga, the lower middle class area, which had the next highest number collected. Aside from the slum, Amorim, where the collection period was shorter than for the other sites, total collections were the lowest in Urca, the upper-middle class area. Mean (SD) weekly *Ae*. *aegypti* collections were the highest in Vila Valqueire (256 ± 74.3), almost double that of Urca (144.1 ± 40.6), which had the lowest collection rate.

**Table 3:** *Ae*. *aegypti* collection data from field sites.

Field site	Total *Ae*. *aegypti* collections	Mean weekly collection ± SD
Vila Valqueire	26625	256 ± 74.3
Tubiacanga	20582	197.9 ± 55.8
Jurujuba	18915	181.8 ± 66.3
Urca	14985	144.1 ± 40.6
Amorim*	1209	75.5± 28.37

*Collection period only 16 weeks

### MRR rate

During the MRR assay, a total of 1,730 *Ae*. *aegypti* females were released in Vila Valqueire. Daily captures occurred for 9 consecutive days after the initial release. A total of 98 marked females were collected during this period for a recapture rate of 5.66%. Of these, 67 (68.4%) were captured with backpack aspirators, while the remainder were recovered from sticky traps. In Jurujuba, 3,000 *Ae*. *aegypti* females were released, with collections again occurring daily for 9 days post-release. We collected 95 marked mosquitoes, corresponding to a recapture rate of 3.17%. Of these, 44 (46.3%) were collected using backpack aspirators, and 51 (53.7%) in sticky traps. The overall recapture rate was much higher for Vila Valqueire than for Jurujuba (Table 4). In Vila Valqueire we observed very similar recapture rates for both Cohorts, however for Jurujuba the recapture rate of Cohort A, where mosquitoes were blood fed prior to release, was almost twice that of Cohort B (Table 4).

**Table 4:** Recapture rate and longevity of *Ae*. *aegypti* females released in Vila Valqueire and Jurujuba.

	Vila Valqueire			Jurujuba		
	Cohort A	Cohort B	Cohort A+B	Cohort A	Cohort B	Cohort A+B
Number of released females	530	1200	1730	900	2100	3000
Recapture rate (%)	5.47	5.58	5.66	4.56	2.47	3.10
PDS by exponential method	0.804	0.721	0.727	0.731	0.839	0.808
PDS by non-linear method	0.819	0.739	0.745	0.744	0.844	0.815
Interval of survivorship	0.77–0.86	0.69–0.80	0.71–0.78	0.68–0.82	0.78–0.91	0.78–0.85
Average life expectancy (days)	3.83–6.63	2.69–4.46	2.86–3.96	2.59–4.95	7.43–10.66	4.07–6.11

Mosquitoes were either blood-fed (Cohort A) or starved (Cohort B) prior to release.

### Daily survival rates and longevity estimates

We used the MRR data to create two models, a non-linear model and an exponential model, in order to estimate the PDS of the two mosquito cohorts that were released at each of the two field sites. The non-linear model provided higher estimates of PDS than the exponential model. This was expected because that model corrects for the removal of mosquitoes collected over previous days (Table 4). Mosquitoes released in the Jurujuba had a higher overall survival rate than those released in Vila Valqueire (Student’s *t* test, *t* = 6.75, *df* = 1, *P* < 0.001). At the latter site, mosquitoes released soon after blood-feeding (Cohort A) had a better survival rate, with the PDS 10.27% higher than for those released under conditions of starvation (Cohort B) (Table 4). However, we observed the opposite pattern in Jurujuba, where the PDS of Cohort B was 13.76% higher than that of Cohort A. We observed variation in estimates of average life expectancy (ALE) between the two field sites, and between the different cohorts released at the same sites. Regardless of the pre-release feeding regime, the expected lifespan of mosquitoes released in Jurujuba was higher than those released in Vila Valqueire, as evidenced by a two-day difference in the ALE upper limit estimate.

### Likelihood of successful *w*Mel invasion at field sites

We used statistical models based on logistic regression that incorporated data from the MRR experiments to estimate the average *Ae*. *aegypti* population size at each of our five field sites (Table 2). The highest estimate of abundance was obtained in Vila Valqueire, with Jurujuba having the second largest population of mosquitoes. The estimated *Ae*. *aegypti* population size at the other three sites was three to four times smaller than either Jurujuba or Vila Valqueire.

**Fig 3:**
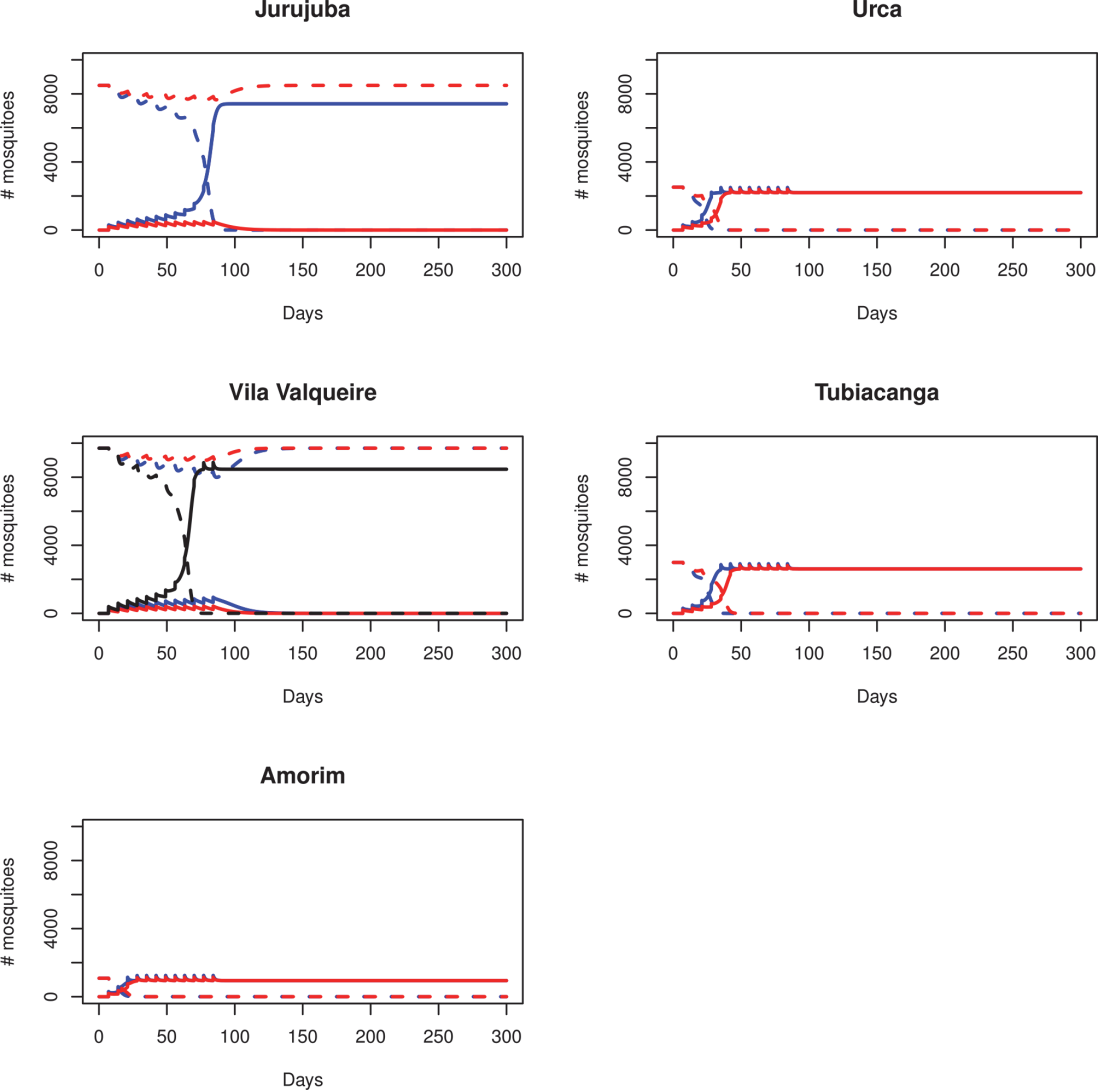
Modeling the invasive potential of *w*Mel at different field sites. Models of the invasive potential of *w*Mel at the five different field sites based on ordinary differential equations assuming a maternal transmission rate of 96% and a longevity cost of 10%. Each plot shows the changes to the total number of *Wolbachia*-infected (solid lines) and—uninfected (dashed lines) mosquitoes (y-axis) in the population over time (x-axis), during the course of a *Wolbachia* invasion involving 12 weekly releases of different sized cohorts of infected mosquitoes. When *Wolbachia* becomes fixed the size of the uninfected population decreases to zero. Red lines depict a weekly release of 200 *Wolbachia*-infected females, which was sufficient for *w*Mel to spread to infect all mosquitoes in Amorim, Tubiacanga, and Urca. Blue lines depict a release of 300 females, which led to successful invasion at all sites except Vila Valqueire, which required 400 females per week (black lines). The differences in the number of mosquitoes at each site reflect the *Ae*. *aegypti* abundance derived from experimental data.

We then used mathematical models based on ordinary differential equations in order to simulate the spread of *w*Mel-infected *Ae*. *aegypti* at each of the five field sites. These models incorporated parameters that were determined from the laboratory experiments and the abundance values derived from the MRR experiments (Table 2). In these models, only the variables of human population size and number of houses were significant predictors. The geographical area and density of inhabitants had no effect. We then compared the effects of different maternal transmission rates and life-shortening levels on the ability of the mosquitoes to spread. The initial scenario assumed a 96% rate of maternal transmission of *w*Mel, and life shortening of 10% associated with infection [7]. We considered scenarios where the numbers of *w*Mel-infected female mosquitoes released each week varied between 200 and 400 (Fig 3). For all five sites, a release number of 200 mosquitoes led to *Wolbachia* becoming established in Amorim, Tubiacanga and Urca inside of 50 days. A release size of 300 was sufficient for the *Wolbachia* infection frequency to reach 100% within 100 days in Jurujuba. For Vila Valqueire the release of 400 female mosquitoes per week was required for complete invasion, and this occurred inside of 100 days.

**Fig 4:**
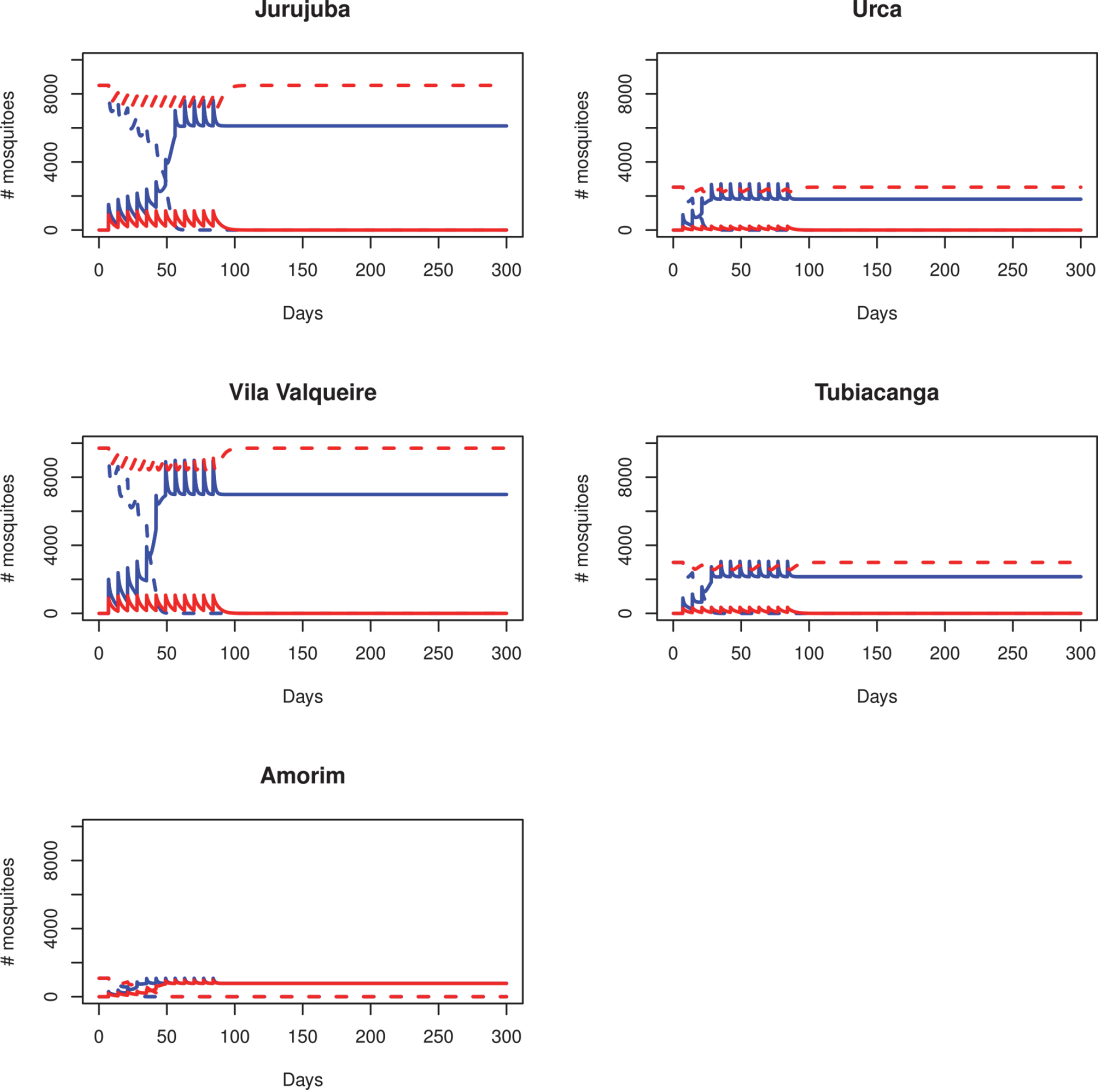
The effect of a stronger life shortening on the invasive potential of *w*Mel. Models depict the invasive potential of *w*Mel with a higher than expected life shortening effect (33% rather than 10%). Each plot shows the changes to the total number of *Wolbachia*-infected (solid lines) and—uninfected (dashed lines) mosquitoes (y-axis) in the population over time (x-axis), during the course of a *Wolbachia* invasion involving 12 weekly releases of different sized cohorts of infected mosquitoes. In this scenario far greater numbers of released mosquitoes were required for *Wolbachia* to reach 100% infection (than for a 10% life shortening effect), and these numbers differed greatly between sites. In Jurujuba, stable infection occurred after weekly releases of 1500 mosquitoes (blue lines), but not 900 (red lines). In Urca, invasion occurred with 900 mosquitoes (blue), but not 200 (red). For Tubiacanga, a release number of 900 mosquitoes was also sufficient to lead to a 100% infection (blue), but 300 was not (red). In Vila Valqueire, releases of 900 mosquitoes was insufficient (red), and 2000 mosquitoes per week were required (blue). In Amorim, releases of both 200 (red) and 300 (blue) mosquitoes led to all mosquitoes becoming infected.

Next, we examined a scenario where the life shortening associated with *w*Mel was greater than expected—increasing from 10% to 33%. Under these conditions the number of mosquitoes required for weekly release increased dramatically (Fig 4). In both Urca and Tubiacanga, the required number was 900 females, with a 100% infection rate being reached inside 50 and 100 days respectively at the two sites. For Jurujuba and Vila Valqueire, which had a greater natural abundance of *Ae*. *aegypti*, the release number required was 1500 and 2000 females respectively, and in both cases, *Wolbachia* was predicted to spread to all mosquitoes inside 100 days. In Amorim, the increased mortality rate had no effect on the number of mosquitoes required, with a 100% infection rate occurring within 50 days under a release regime of 200 or 300 mosquitoes.

We then considered the effect of a higher maternal transmission rate (99%) for both of the above scenarios. When life shortening was 10%, the increased maternal transmission rate had no effect on the spread of *Wolbachia* at Tubiacanga or Urca (S1 Fig). For Amorim, the decreased fitness cost led to equivalent invasion profiles with either 200 or 300 females per week. However, at both Jurujuba and Vila Valqueire, the number of released mosquitoes required to achieve stable infection was decreased, and the time to achieve a stable infection was shorter. When life shortening was increased to 33%, the increased maternal transmission did not affect the release dynamics at any of the field sites, with cohorts of similar size to those at the lower maternal transmission rate required to increase the frequency of *Wolbachia* infection (S2 Fig).

## Discussion

### Characterisation of *w*Mel infection in Brazilian *Ae*. *aegypti*

The use of *Wolbachia* to control dengue transmission represents a novel and promising method of mosquito biocontrol. Although the technique is still in early-stage field trials and restricted to small or isolated areas in a handful of countries, the scope and scale of these trials will inevitably expand. Releases of *Ae*. *aegypti* infected with the *w*Mel *Wolbachia* strain have recently commenced in Brazil, where regulatory agencies provided approval for releases at four isolated sites through 2016 (these being four of the field sites examined in this article, with the exception of Amorim). Given that there could potentially be great variability in the conditions present at these sites, it is important to understand how differences in physical characteristics, human population demographic factors, and local mosquito population characteristics between these sites might affect the likelihood of successful invasion by *Wolbachia*. If there was such an effect, it would necessitate that key components of the release process (e.g. mosquito release numbers, number of release points, release period length) be tailored to individual release sites.

For *Wolbachia* to successfully invade in a given area, the infected mosquitoes must display a high level of cytoplasmic incompatibility [5]. This would compensate for imperfect maternal transmission of *Wolbachia* and any detrimental effects on host fitness resulting from infection, if either of those things were to occur [29]. In the case of the Brazilian releases, it was first necessary to transfer the *w*Mel strain to mosquitoes with a Brazilian genetic background. Our results indicated that near-complete cytoplasmic incompatibility occurred when infected males mated with uninfected females. This would provide infected mosquitoes with a large reproductive advantage over uninfected mosquitoes in a mixed population, as might occur during a field release. Interestingly, we observed that egg viability was higher when uninfected males mated with uninfected females than with *Wolbachia*-infected females. This suggests that there may be a small cost associated with *w*Mel infection that lead to reduced egg viability. When mated with uninfected males, the majority of *Wolbachia*-infected females displayed an egg hatch level that was comparable to that produced by matings of uninfected males and females, while a small group of these infected females laid eggs with greatly reduced viability. It is possible that this group of females all had higher than normal levels of *Wolbachia*, as this can lead to more extreme phenotypes in the host [30].

Fecundity is an important life-history trait in invertebrates, and is also important for *Wolbachia*-based control strategies, as higher fecundity will promote a more rapid spread of the bacterium [31]. In our experiments, we observed no significant difference in fecundity levels due to the presence of the *w*Mel infection, which suggests that there will be no fitness impact on this trait due to infection. This was similar to what was observed in *w*Mel-infected *Ae*. *aegypti* with an Australian genetic background [7].

In one of two experiments, we detected a lower rate of *w*Mel maternal transmission in the Brazilian genetic background than what has previously been described (96% as opposed to 100% in the other experiment) [6,7]. Loss of transmission efficiency can be associated with environmental factors like high temperature [32], or with the host genetic background [33]. Given the consistency of laboratory temperatures, a difference due to the change in host genetics represents a more plausible scenario. Another potential explanation could be due to the *Ae*. *aegypti*-*Wolbachia* relationship, which is still rather novel from an evolutionary point of view. Consequently, we would expect a stronger host immune response to *Wolbachia* [34,35], which could potentially promote the loss of infection in some individuals [36]. This could occur if the immune response of Brazilian mosquitoes to *Wolbachia* is different to that of other clades. It is also possible that the maternal transmission rate could have been underestimated, and some of the samples were falsely identified as negative, perhaps due to insufficient dilution prior to qPCR at that time. Given the high levels of maternal transmission that have previously been observed for *w*Mel [7], we were of the opinion that the results of the second experiment were more likely to be accurate. Nevertheless we also included the lower rate in the subsequent models.

### Characterization of local mosquito populations

The ability of *Wolbachia* to become fixed in wild mosquito populations has previously been examined using theoretical models [37]. However, none of these have considered how conducting the experiments in large, heterogeneous cities such as Rio de Janeiro would affect the likelihood of a *Wolbachia* strain spreading effectively. *Ae*. *aegypti* life history traits and population size may vary dramatically between districts, depending on the nature and influence of urban landscapes and human demographic factors. This in turn would influence how *Wolbachia* might spread. To test these effects, we sought to characterise five distinct field sites in Rio de Janeiro, and the mosquito populations dwelling within, and then used mathematical models to predict how the resulting differences might affect invasion by *Wolbachia*.

By conducting mosquito collections over a period of two years, we were able to make estimates of existing mosquito population sizes, which varied greatly amongst our five field sites. The average weekly mosquito collection did not correlate with the development index of the sites. Of the four sites where collections ran the full 104 weeks, Vila Valqueire had the highest collection rate, and Urca the lowest. Interestingly, these two sites had the highest developmental index of our five sites. Both sites contain large houses/duplexes, have piped water, and organized garbage collection and have good mosquito control programs. This suggests that the developmental index of these areas is not a major determinant of the number of mosquitoes at these sites. In Urca there was no regular use of external water tanks, which may also have contributed to the lower prevalence of *Ae*. *aegypti* in the area.

In our MRR experiments we observed that the mosquito recapture rate was approximately twice as high for Vila Valqueire as for Jurujuba. This could have occurred because of increased access to properties in Vila Valqueire as a result of greater assistance from local residents. Alternatively, it is possible that insecticide usage may have been higher in Jurujuba during the experiment. During this experiment we released two different cohorts of mosquitoes, one blood fed prior to release, and the other unfed. The bloodfed mosquito cohort (A) was recaptured at a far greater rate than the non-bloodfed cohort (B) in Jurujuba, but there was no difference in the recapture rate between the two cohorts at Vila Valqueire. It is possible that this difference in recapture rate occurred due to a specific micro-environmental effect in Jurujuba that differentially affected the two cohorts, however it is not currently clear what that might be. Alternatively, this observation could have been an artefact of small collection numbers, rather than a real difference. We also observed a differential effect of pre-release blood feeding on survival, with the PDS of Cohort A mosquitoes higher than Cohort B in Vila Valqueire, but lower in Jurujuba. This suggests that there may certain conditions present at some field sites where it would be advantageous to release blood fed individuals. Similar experiments should be conducted with *w*Mel-infected mosquitoes to determine if this effect is consistent in the presence of *Wolbachia*.

Comparable MRR experiments for the other three field sites; Amorim, Urca and Tubiacanga had been performed previously [19,22]. In these experiments only non-blood fed females were released, so the results were comparable to our data for Cohort B. Overall, we observed a much lower recapture rate for Jurujuba and Vila Valqueire than what was previously described at the other sites. A comparison of all sites revealed the highest recapture rates were in Amorim (13.10%) and Tubiacanga (12.82%) during the wet season. These values decreased slightly during the dry season but were still higher than either of the two new sites, as was the recapture rate in Urca (9.77%). The ALE of mosquitoes at Vila Valqueire was similar to those from Urca and Tubiacanga (during the wet season). While ALE values at Jurujuba were similar to those from Amorim (during both the wet and dry seasons) and Tubiacanga (during the dry season).

We observed great variation in the daily survival rate of mosquitoes between our five field sites. The highest PDS (0.93) was observed in the less affluent district of Amorim [22], while the lowest PDS of 0.60 was observed in Urca, which had the highest level of social development. The PDS of the other three sites ranged between 0.72 and 0.81. Although we obtained data for only five sites, we saw a very strong negative correlation between field site affluence and mosquito PDS. This suggests that the developmental area of a particular area could potentially have some effect on the survival of local mosquitoes, which is likely reflective of a disparity in mosquito control measures. We also saw a strong positive correlation between human density and PDS, which could be explained by the fact that higher density makes it easier for mosquitoes to find hosts and feed successfully. The fact that there were such considerable differences in PDS between the sites indicates that this is a factor that must be considered when planning releases. A lower PDS coupled with a large wild population could make it more difficult for *Wolbachia*-infected mosquitoes to become established, especially taking into account potential life shortening effects due to infection.

### Invasive potential of *w*Mel-infected Brazilian *Ae*. *aegypti*

We utilised the data obtained through the laboratory and field experiments described above to model the spread of *w*Mel through the five field sites. Our models indicated that two field sites, Jurujuba and Vila Valqueire, had far greater abundance of *Ae*. *aegypti* than the other three sites. There are several potential explanations for the differences we observed in mosquito abundance. There could have been differences in the numbers, location, and favorability in breeding sites, differences in the likelihood of finding a host, differences in mosquito control measures, or in environmental conditions. All of these factors could not be directly considered in the statistical models. Interestingly, our data did not indicate that there was a strong relationship between PDS and abundance. Vila Valqueire and Jurujuba had high abundance but far lower numbers of houses and inhabitants that the other three field sites. This is somewhat counterintuitive, as a positive correlation between abundance and these factors makes more sense when considering the biological requirements of mosquito populations for hosts and breeding sites. Given that there are a large number of factors that affect the size of a mosquito population, some of which have not been considered in our models, it is unclear if this effect is something real or an artifact of considering only five locations.

Estimates of abundance at each of the five sites were directly incorporated in our models, and used to represent the initial size of *Wolbachia*-uninfected mosquitoes naturally present at each site prior to the time of release. The release protocols used in these models were simulated based on the general format of *Wolbachia*-infected mosquito releases as utilised as part of the Eliminate Dengue Project [11]. This involves the weekly release of a number of *Wolbachia*-infected female mosquitoes for a period of several months, during which time the frequency of *Wolbachia*-infected individuals in the population typically rises and then eventually reaches 100%.

Our models indicated that the *w*Mel infection frequency would likely reach 100% at each of the five field sites, with only a small number of mosquitoes required for release each week. Interestingly though, the requirements for a successful release differed between the sites in terms of the numbers of mosquitoes required, and the expected period of time required for *Wolbachia* to become fixed. Unsurprisingly, these differences proved to be strongly related to the predicted mosquito abundance at the sites. Under the initial modelled conditions of a low cost of infection and slightly decreased maternal transmission rate, we determined that a weekly release of 200–300 females was typically sufficient for a successful invasion for all sites with the exception of Vila Valqueire, which had the greatest abundance and mosquito carrying capacity of all the sites. This represented a release cohort far smaller than what was released in Australia [11]. For Amorim, Tubiacanga and Urca, which all had smaller wild mosquito populations, the models predicted that the infection would spread to all mosquitoes in the area within the first 50 days after the initial release occurred. These results were expected, given the high importance of mosquito abundance on the ability of *Wolbachia* to spread quickly [12]. Even so, a period of less than 100 days was predicted for the two sites with higher abundance. The models indicated that a lower maternal transmission rate was a large determining factor in the dynamics of invasion, as an increase in the modelled rate from 96% to 99%, led to a decrease in the number of mosquitoes and time required for the *Wolbachia* infection to spread to 100%.

The success of *Wolbachia*-infected mosquitoes in all of these locations is strongly linked to fitness costs resulting from the infection [11,38]. While the cost associated with *w*Mel infection is fairly low [11] we also considered a scenario where a stronger than expected life shortening effect occurred amongst released mosquitoes. This resulted in less effective invasion. At the three sites with lower abundance, the number of mosquitoes required for weekly release tripled, while at the sites with higher abundance, five times more mosquitoes were required. These numbers may appear high, but they are still significantly lower than the what was released each week at field sites in Australia [11]. Our models also indicated that the detrimental effect of this fitness cost on invasive potential was far greater than the benefit obtained through a higher maternal transmission rate, as a combination of these two parameters led to invasion patterns that were largely similar to those seen with the lower maternal transmission rate. This scenario could be considered a worst-case scenario for invasion with *w*Mel, and is also rather unlikely to eventuate given that such extreme fitness effects have not been observed in *w*Mel-infected mosquitoes after more than two years in the field [38]. What is encouraging is that, although the invasion was less effective under these conditions, our data suggest that it is still possible for *Wolbachia* to spread and completely infect the mosquito population at any of the five sites.

The most important factor influencing the spread of *Wolbachia* at any of the field sites was local mosquito abundance, and our data reinforce the need to release *Wolbachia*-infected mosquitoes in higher numbers at sites where there are larger populations of wildtype mosquitoes [28,29,37]. This factor is clearly more of an influence than any of the urban landscape conditions that we analysed. However there is little doubt that there are socio-demographic factors that are important to determining abundance. For instance a larger human population and greater human density would likely provide a higher chance of locating a blood meal host, and potentially an increase in the number of favourable breeding site containers. Better sanitation conditions at the field site would be expected to have the opposite effect, decreasing breeding site availability. We observed the greatest abundance of *Ae*. *aegypti* at Vila Valqueire and Jurujuba. These sites had by far the smallest number of houses and total human population of the five sites. They differed from each other in terms of sanitation (no garbage collection in Jurujuba), and human density (high in Jurujuba, but low in Vila Valqueire). It is difficult to establish relationships between these variables and abundance, and part of the reason may be that they are only indirect or partial determinants. A similar study in Australia, focusing more specifically on micro-environmental conditions at the residential block level, indicated that the type of house, level of shade on the property, and availability of breeding sites all affected the proportion of mosquitoes in the area that were infected with *Wolbachia* [39]. Our study was not designed to assess differences between sites at this level, however there is likely a strong link between these parameters, and those from our models.

There were some factors that were not considered in our models that could potentially contribute to differences in wildtype mosquito abundance between the sites. The first of these is that seasonal differences were not taken into account. Estimations of mosquito population size at each site were an average taken across all collection points, and normalized the effects of time and spatial homogeneity across the area. In addition, the MRR data, which was utilised to calculate abundance, were obtained from experiments conducted over a comparatively shorter period of time, and could have been influenced by seasonal effects. As the MRR experiments at Jurujuba and Vila Valqueire were conducted separately to those for the other sites, this could also have contributed to the higher abundance and lower recapture rates observed there. The data obtained from Amorim were also interesting. There we observed the highest PDS of all the sites, yet the carrying capacity was the lowest, and the site was the easiest of the five for *Wolbachia* to invade. It is possible that given the shorter mosquito collection period at this site, the carrying capacity was underestimated, or perhaps the mosquito population is just naturally low at that location in spite of the high human density. Given the complexity and heterogeneity of the landscapes encompassing our field sites it is entirely possible that there are other unmeasured or potentially cryptic factors that could contribute to local mosquito abundance or to the successful spread and invasion of *Wolbachia*. It is also possible that, given the developing nature of the field sites, and other future release areas in different countries there may be changes in the landscape over time that may have a similar effect. To that end, it would be useful to consider future modelling at these or other sites in the context of high resolution, real-world landscape data, as such models would be both more rigorous and more sensitive [40,41]. At the time of this study such data were not available, and while they would have no doubt proven to be valuable, they were too costly to obtain.

### General conclusions

Our results suggest that the *w*Mel strain of *Wolbachia* will likely be able to successfully invade five distinct field sites around the city of Rio de Janeiro, Brazil. We observed both strong CI, and no decrease to fecundity associated with infection, both of which will promote invasion. Our survival data indicated that there were differences in the daily probability of survival for mosquitoes at the different sites, with the better-developed sites being less hospitable. We also observed that *w*Mel was able to spread more effectively when the local mosquito population was smaller. This suggests that *Wolbachia* could potentially struggle to spread at better-developed sites that have more abundant mosquito populations, as we observed with Vila Valqueire. Critically, although we saw a difference in the invasive potential of *w*Mel between the field sites, the maximum numbers of mosquitoes required for weekly releases was only 2000, and under most conditions, the number was far lower. These data support the hypothesis that releases would be more effective during the dry season, when the mosquito population is naturally smaller [11].

We show that field sites in Rio de Janeiro are complex landscapes that support mosquito populations of greatly different sizes, and this can affect the dynamics of *Wolbachia*. However, inter-site differences in key aspects of these landscapes do not serve as an impediment to the overall success of invasion by *w*Mel. In spite of this it can be beneficial to adequately characterise field sites prior to the release of *Wolbachia*-infected mosquitoes as this will help determine the optimal release strategy. Our results provide a useful framework for the ongoing field-releases of *w*Mel-infected *Ae*. *aegypti* in Brazil, and will likely hold similar utility for future releases of these mosquitoes in other similarly heterogeneous urban landscapes.

## Supporting Information

S1 Table Raw capture data from the Mark-Release-Recapture experiments in Jurujuba and Vila Valqueire. Marked mosquitoes were released into the field sites and then re-captured over the course of 9 days using both backpack aspirators and Sticky traps. Marked mosquito numbers represent both Cohorts A and B (see methods section for details). Unmarked mosquitoes were not released as part of the experiments.
(DOCX)

S1 Fig Modeling the invasive potential of *w*Mel with a 99% vertical transmission rate. Under this scenario the maternal transmission rate of *w*Mel increased to 99%, while the life shortening effect remained at 10%. All modeled releases sizes were as per Fig 3 (red; 200 females, blue; 300, black; 400). In this case a release cohort size of 300 led to a Wolbachia infection frequency of 100% at all sites. In general, invasion occurred more quickly at all sites after the initial release than with a maternal transmission rate of 96%.
(TIF)

S2 Fig Modeling the invasive potential of wMel with a 99% vertical transmission rate, and increased life shortening. The models here depict the invasive potential of wMel with the maternal transmission rate increased to 99% and the life shortening effect increased to 33%. Mosquito release numbers were the same as those depicted in the models from Fig 4. The models here indicate that the adverse effects of increased life shortening, which necessitate the release of additional mosquitoes each week, are not ameliorated by the increase in vertical transmission. As such the dynamics of wMel invasion were not different from the data presented in Fig 4.
(TIF)
