# Supplementary material for: From Lab to Field: The Influence of Urban Landscapes on the Invasive Potential of Wolbachia in Brazilian Aedes aegypti Mosquitoes
Source: PLoS Negl Trop Dis. 2015 Apr 23;9(4):e0003689. doi: 10.1371/journal.pntd.0003689 (PMC4408005; doi:10.1371/journal.pntd.0003689)
Supplement: S1 Table — Marked mosquitoes were released into the field sites and then re-captured over the course of 9 days using both backpack aspirators and Sticky traps. Marked mosquito numbers represent both Cohorts A and B (see methods section for details). Unmarked mosquitoes were not released as part of the experiments. (DOCX) [file pntd.0003689.s001.docx]

**Supplementary Table 1:** Number of marked and unmarked mosquitoes collected in Vila Valqueire and Jurujuba using backpack aspirator and sticky traps during the MRR experiment.

|  | **Vila Valqueire** | | | | **Jurujuba** | | | |
| --- | --- | --- | --- | --- | --- | --- | --- | --- |
|  | Backpack aspirator | | Sticky trap | | Backpack aspirator | | Sticky trap | |
| **Day** | Marked | Unmarked | Marked | Unmarked | Marked | Unmarked | Marked | Unmarked |
| 1 | 24 | 38 | 4 | 12 | 12 | 4 | 9 | 6 |
| 2 | 12 | 70 | 6 | 7 | 9 | 8 | 12 | 1 |
| 3 | 9 | 24 | 5 | 9 | 4 | 12 | 10 | 3 |
| 4 | 5 | 35 | 5 | 5 | 8 | 5 | 6 | 1 |
| 5 | 11 | 47 | 4 | 3 | 2 | 3 | 4 | 2 |
| 6 | 3 | 24 | 3 | 9 | 3 | 3 | 3 | 2 |
| 7 | 2 | 58 | 2 | 10 | 3 | 1 | 1 | 5 |
| 8 | 0 | 56 | 1 | 4 | 2 | 6 | 3 | 5 |
| 9 | 1 | 57 | 1 | 7 | 1 | 6 | 3 | 6 |
| **Total** | **67** | **409** | **31** | **66** | **44** | **48** | **51** | **31** |
