# Supplementary figures and images for: From Lab to Field: The Influence of Urban Landscapes on the Invasive Potential of Wolbachia in Brazilian Aedes aegypti Mosquitoes
Source: PLoS Negl Trop Dis. 2015 Apr 23;9(4):e0003689. doi: 10.1371/journal.pntd.0003689 (PMC4408005; doi:10.1371/journal.pntd.0003689)

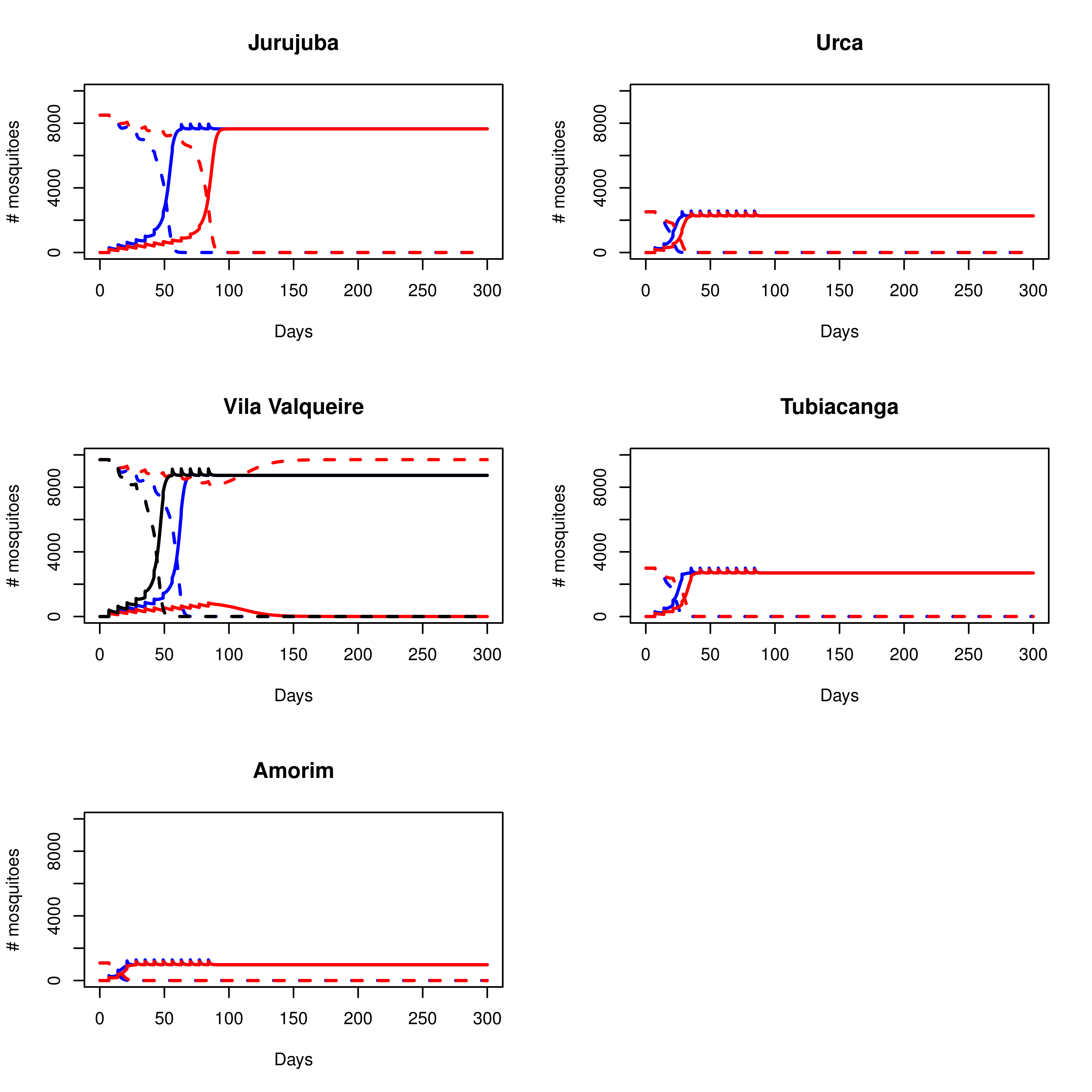

Supplement: S1 Fig — Under this scenario the maternal transmission rate of wMel increased to 99%, while the life shortening effect remained at 10%. All modeled releases sizes were as per Fig 3 (red; 200 females, blue; 300, black; 400). In this case a release cohort size of 300 led to a Wolbachia infection frequency of 100% at all sites. In general, invasion occurred more quickly at all sites after the initial release than with a maternal transmission rate of 96%. (TIF) [file pntd.0003689.s002.tif]

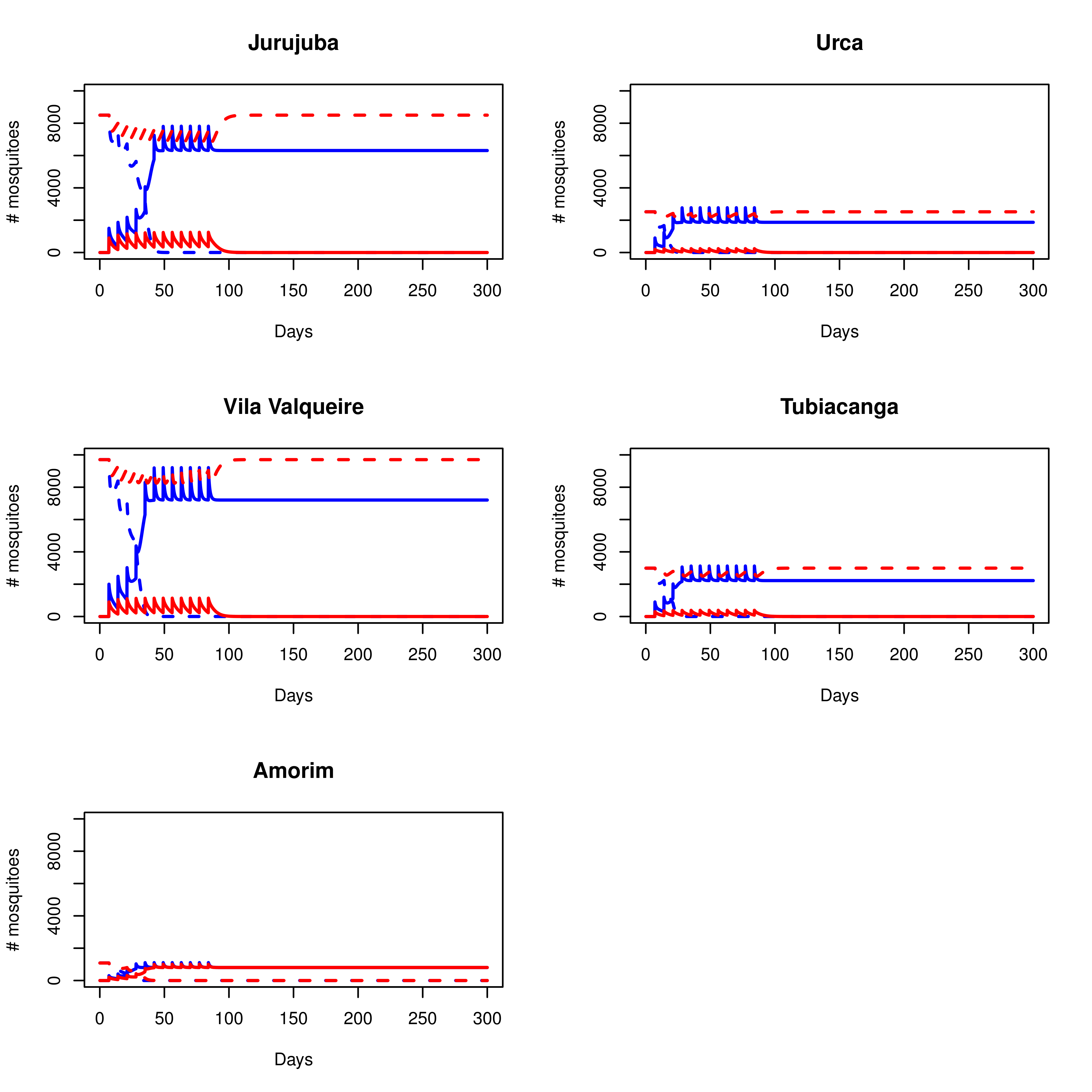

Supplement: S2 Fig — The models here depict the invasive potential of wMel with the maternal transmission rate increased to 99% and the life shortening effect increased to 33%. Mosquito release numbers were the same as those depicted in the models from Fig 4. The models here indicate that the adverse effects of increased life shortening, which necessitate the release of additional mosquitoes each week, are not ameliorated by the increase in vertical transmission. As such the dynamics of wMel invasion were not different from the data presented in Fig 4. (TIF) [file pntd.0003689.s003.tif]
